# Host resistance responses against *Puccinia striiformis* f. sp. *tritici* in wheat cultivars with different resistance levels: molecular, biochemical, and ultrastructural studies

**DOI:** 10.1186/s12870-024-05811-0

**Published:** 2024-11-28

**Authors:** Hany H. A. El-Sharkawy, Younes M. Rashad, Zakaria A. M. Baka, Adel K. Madbouly, Osama E. Abd El Badeea

**Affiliations:** 1 Mycology Research and Diseases Survey Department, Plant Pathology Research Institute, Agricultural Research Center, Giza, Egypt; 2 Plant Protection and Biomolecular Diagnosis Department, Arid Lands Cultivation Research Institute (ALCRI), City of Scientific Research and Technological Applications (SRTA-City), New Borg El-Arab City, 21934 Egypt; 3 Department of Botany and Microbiology, Faculty of Science, Damietta University, New Damietta, Egypt; 4 Microbiology Department, Faculty of Science, University of Ain Shams, Abbassia, Cairo, Egypt; 5 Wheat Diseases Research Department, Plant Pathology Research Institute, Agricultural Research Center, Giza, Egypt

**Keywords:** Haustoria, Stripe rust, *Triticum aestivum*, Plant resistance, *Yr* genes

## Abstract

**Background:**

Yellow (stripe) rust of wheat, caused by *Puccinia striiformis*, is a serious disease that results in great economic losses. This study aimed to investigate the variation in plant responses in three wheat cultivars with different resistance levels against yellow rust.

**Results:**

The highest disease severity was recorded for *cv*. Gemmieza-11 (95%), followed by *cv*. Shandweel 1 (60%), while the lowest was recorded for *cv*. Misr-3 (3%). qPCR results of the yellow rust-resistance genes *Yr5*, *Yr10*, *Yr15*, and *Yr18* showed that the infection did not affect the expression of *Yr5* and *Yr15* in the infected Gemmieza-11 plants when compared to the non-infected control. In contrast, the infection significantly overexpressed *Yr5* and *Yr15* in *cvs*. Shandweel 1 and Misr-3. However, Misr-3 was superior in this regard, recording 3.85- and 4.07-fold for *Yr5* and *Yr15*, respectively. In addition, the infection significantly upregulated *Yr10* and *Yr18* in the three tested cultivars, with the superiority for the cultivar Misr-3, followed by Shandweel 1. Activity of the antioxidant enzymes peroxidase, polyphenol oxidase, and catalase was significantly higher in the infected plants of *cv*. Misr-3 than in *cv*. Shandweel-1, while the lowest values were recorded in *cv*. Gemmieza-11. The increment in this activity was associated with a reduction in the lipid peroxidation in the three tested cultivars. The phenolic content considerably increased also upon infection in *cv*. Misr-3 followed by Shandweel-1 but not in *cv*. Gemmieza-11, compared to the non-infected plants. Transmission electron microscopy demonstrated that the infected mesophyll cells in *cv*. Gemmieza-11 showed abnormalities in the chloroplasts and thick-walled haustoria. On the contrary, the mesophyll cells in *cv*. Misr-3 showed no haustoria and well organized chloroplasts. The mesophyll cells in *cv*. Shandweel-1 displayed highly degenerated haustoria, a degenerated granulated cytoplasm, and a thick host cell wall, indicating intermediate defense responses against the invading pathogen. Plant growth, yield, and photosynthetic pigments were higher in *cv*. Misr-3, followed by *cv*. Shandweel-1, and the lowest values were recorded in *cv*. Gemmieza-11.

**Conclusions:**

The obtained results displayed that *Yr5* and *Yr15* were more effective than *Yr10* and *Yr18*, indicating their main roles in regulating multiple defense mechanisms and hypersensitive responses.

**Supplementary Information:**

The online version contains supplementary material available at 10.1186/s12870-024-05811-0.

## Background

Wheat yellow rust, known as stripe rust, is caused by the biotrophic fungus *Puccinia striiformis* f. sp. *tritici* Erikss. (Phylum Basidiomycota). *Puccinia striiformis* is one of the most prevalent and destructive threats in most wheat (*Triticum aestivum* L.) growing regions [1]. Gadd (1777) described this disease for the first time on rye; however, the pathogen was first identified in 1854 by Westendorp as *P. striaeformis.* In 1896, Eriksson and Henning renamed the pathogen as *P. glumarum*, which was later changed to *P. striiformis* by Hylander et al. in 1953 [2].

This fungus attacks cereal crops, mainly wheat and barley; however around 300 wild grasses from 50 genera can harbor the pathogen as well [3]. *Puccinia striiformis* has a heteroecious and macrocyclic lifecycle, where five types of spores (uredospores, teliospores, basidiospores, pycnidiospores, and aeciospores) occur on two alternative hosts, a cereal crop or a grass as the main host and an alternate host plant such as *Berberis* spp. [4]. Uredospores can be carried by wind and migrate for long distances. Under appropriate weather conditions (saturated moisture at around 10 ºC), the uredospores land on a wheat leaf, rapidly germinate on the adaxial leaf surface, form a germ tube with a penetration peg within 5 h, and penetrate the leaf through the stomatal cavity into the intercellular spaces of the mesophyll layer. Afterward, they absorb nutrients and water from the leaf cells *via* their haustoria, which are formed inside the cells [5]. The young haustorium has a spherical shape, while the older one is more branched to increase the contact area and nutrients uptake [6]. After the incubation period, oval to spindle-shaped yellow uredial pustules are formed in the epidermal layer causing the appearance of parallel yellow-colored stripes along the host leaf. These uredial pustules erupt at their maturity, thousands of uredospores are released, and the uredial pustules are converted into black colored telia containing teliospores [7]. The produced teliospores can survive during winter and infect the alternate host when the weather conditions are favorable.

In Egypt, there are several wheat cultivars showing increasing resistance levels to yellow rust; mainly Gemmieza-11, Shandweel-1, and Misr-3, respectively. A previous study conducted by Mabrouk et al. [8] during two growing seasons reported that *cv*. Gemmieza-11 had the highest rates of yellow rust increase, final rust severity, actual yield losses for 1000 kernel weight/g, grain yield/plot, and area under disease progression curve (AUDPC), while the moderately resistant *cv*. Shandweel-1 displayed moderate levels of these tested parameters in addition to lower yield losses. Abdel-Majeed et al. [9] demonstrated that *cv*. Misr 3 was released by the ARC, Egypt, in 2016, and offered the wheat growers a choice for high yield and development of a new rust resistant wheat genotype. Furthermore, Draz et al. [10] revealed higher levels of pleiotropic adult plant resistance observed against the stem, leaf, and stripe rusts in nine wheat cultivars; mainly *cv*. Misr-3. In a previous study, Abu Aly and Abd El-Kreem [11] tested fifteen crosses including *Yr5*, *Yr10*, and *Yr15* and the wheat cultivars Sakha 93, Gemmieza-10, Gemmieza-11, Sids 12, and Sids 13. The study showed that wheat cultivars which had *Yr5*, *Yr10*, and *Yr15* genes were resistant and showed low stripe rust reaction and severity at the seedling and adult stages, respectively. Meanwhile, Hagras et al. [12] study revealed that the efficiency of stripe rust resistance genes can be arranged in the order of *Yr15>Yr27>Yr8>Yr57>Yr34* with Shandaweel-1 and Gemmeiza-11 background. Recently, Shahin et al. [13] suggested that wheat breeding programs can utilize *Yr* genes representing effective resistance genes to develop novel genotypes with stripe rust resistance. The *Yr5* and *Yr10* genes were recently introgressed into a number of susceptible genotypes, including Gemmieza-11, Misr1, and Misr2, which are widely grown and recognized as sources of superior flour.

Due to their long-lasting residue levels, improper usage of the synthetic pesticides leads to severe environmental pollution. Management of yellow rust involves usage of integrated control strategies; mainly resistant cultivars, induced resistance, fungicide application, and cultural and bio-control methods [3]. Many studies have been conducted to control yellow rust using different techniques [14–16]. However, cultivating resistant cultivars of wheat is the most economic and effective strategy to control this pathogen. In this regard, many yellow rust-resistance genes (*Yr*) have been identified till date, including *Yr5*, *Yr10*, *Yr15*, *Yr18*, and *Yr5/ YrSP* [17]. Despite the high and variable numbers of the identified rust-resistance genes, just few of them are still effective due to the rapid and continuous evolution of *P. striiformis*, which results in the emergence of new highly pathogenic races of this fungus around the world [18].

Reactive oxygen species (ROS) usually accumulate in plants suffering from biotic stresses. Moreover, it has been found that production of ROS is critical for the rust-fungus virulence and germination of its uredospores [19]. On the contrary, upon exposure of wheat plants to yellow rust, they tend to protect themselves *via* activating multiple effective antioxidant systems, including antioxidant metabolites and enzymes; mainly peroxidase (POD), polyphenol oxidase (PPO), 1,3-glucanase, and chitinase [20]. Different defense mechanisms have been reported against yellow rust of wheat, including stimulation of different signaling pathways, activation of antioxidant enzymes, overexpression of some genes of pathogenesis-related (PR) protein, accumulation of several phenolic compounds, and lignin deposition at the infection sites [21, 22]. In the recent study reported by Rashad et al. [23], a considerable upregulation of polyphenols biosynthesis genes took place in wheat against yellow rust pathogen upon treating with the endophyte *Epicoccum nigrum* HE20. The objectives of this study were to compare the plant-pathogen interactions and host defense responses against *P. striiformis* in resistant, moderately susceptible, and susceptible wheat cultivars based on molecular, biochemical, and ultrastructural levels.

## Methods

### Wheat cultivars and the pathogenic fungus

During this study, grains of wheat cultivars with different background resistance to yellow rust; *cv*. Gemmieza-11 (susceptible), Shandweel-1 (moderately susceptible), and Misr-3 (resistant) were used, which were provided by the Crop Research Institute, Agricultural Research Center (ARC), Giza, Egypt. Fresh and aggressive uredospores of *P. striiformis* f. sp. *tritici* (race 151E80) were obtained from the Wheat Diseases Research Department, Plant Pathology Research Institute (PPRI), ARC, Egypt. Using sterile water, 0.1% tween 80, and gum Arabic powder (40 gL^− 1^), a suspension of uredospores (10^4^ spore mL^− 1^) was prepared.

### Greenhouse experiment

Surface-sterilized grains (using 5% sodium hypochlorite) of the wheat cultivars Gemmieza-11, Shandweel-1 and Misr-3 were planted in 30 cm diameter pots (at 10 grains per pot), which were filled with sterile soil (sand/clay 3:1, v/v). At the time of planting and 45 days later, NPK fertilization was applied two times at 1: 1.5: 0.5 g pot^− 1^. Fifty-five days after planting (stage of spike formation), wheat plants from each cultivar were fully sprayed with a freshly prepared uredospores suspension. As a control treatment and for each cultivar, the pots were sprayed with sterile water only. Using tap water, the pots were irrigated to near the field capacity. Fifteen pots were applied for each treatment. The pots were arranged in a complete randomized design. To simulate the infection, the pots were kept at 10–12 °C and 90% humidity in a transparent plastic hood for 48 h, and then placed in a greenhouse at 17/23°C (day/night), 60% humidity, and a 16 h light period. The applied treatments included: non-infected Gemmieza-11 (G11), infected Gemmieza-11 (G11 + P), non-infected Shandweel 1 (S1), infected Shandweel 1 (S1 + P), non-infected Misr-3 (M3), and infected Misr-3 (M3 + P).

### Yellow rust evaluation

Fifteen days post inoculation (dpi), and according to a diagrammatic scale (1-100%) designed by Peterson et al. [24], the severity of yellow rust was evaluated. The average coefficient of infection (ACI) was estimated by multiplying the value of severity by the value of infection type as described by Saari and Wilcoxson [25]. Twelve plants of each treatment were evaluated.

### Quantitative expression of the yellow rust-resistance genes

Four dpi, leaf samples from the tested treatments were collected. To extract the total RNA from the collected samples, a PureLink RNA Mini Kit (Invitrogen, Carlsbad, CA, USA) was used according to the supplied manual. cDNA was synthesized from each RNA sample using ProFlex thermal cycler (Thermo Fisher Scientific, San Francisco, CA, USA). The PCR mixture (20 µL) contained 3.5 µL RNA (200 ng µL^− 1^), 4.5 µL oligo (dT) primer (20 pmol µL^− 1^), 3 µL dNTPs (10 mM), 3 µL 5X buffer, 0.3 µL RT enzyme (50 U µL^− 1^, Omniscript RT, Qiagen, Germany), and 5.7 µL RNase-free water. The PCR thermocycler protocol was carried out as follows; 40 °C for 70 min, 95 °C for 12 min.

Real-time quantitative PCR (qPCR) was conducted using LightCycler^®^ 480 Real-Time PCR system (Qiagen, USA) to quantify transcriptional expression of the yellow rust-resistance genes (*Yr5*, *Yr10*, *Yr15*, and *Yr18*) in each cultivar [26]. The qPCR mixture (20 µL) included cDNA (2.5 µL, 20 ng), primer (F) (1.5 µL, 10 pmol µL^− 1^), primer (R) (1.5 µL, 10 pmol µL^− 1^), SYBR Green PCR Master Mix (10 µL, Bioline, Germany), and nuclease-free water (4.5 µL). The used primers are presented in Table 1. The qPCR protocol was performed as follows; 95 °C/ 2.5 min, 40 cycles (95 °C/ 20 s, 56 °C/ 30 s, and 73 °C/50 sec). To estimate the gene expression, a comparative CT method (2^−∆∆CT^) was applied [27]. For each sample, 3 biological and 3 technical replicates were used.

**Table 1:** Sequence of primers used in this study

Gene name	Primer name	Sequence (5′-3′)
*Yr5*	*Xwmc175*-F *Xwmc175*-R	GCTCAGTCAAACCGCTACTTCT CACTACTCCAATCTATCGCCGT
*Yr10*	*Yr10R1*-F *Yr10R1*-R	TTGGAATTGGCGACAAGCGT GTGATGATTACCCACTTCCTC
*Yr15*	*XBarc8*-F XBarc8-R	GCGGGAATCATGCATAGGAAAACAGAA GCGGGGGCGAAACATACACATAAAAACA
*Yr18*	*Cslv34*-F	CTGGTTAAGACTGGTGATGG
	*Cslv34*-R	TGCTTGCTATTGCTGAATAGT
*β-actin*	*β-actin*-F	GTGGGCCGCTCTAGGCACCAA
	*β-actin*-R	CTCTTTGATGTCACGCACGATTTC

### Effect of infection on the biochemical defense responses

Three dpi, leaf samples from the tested cultivars were collected and subjected to different biochemical analyses. The antioxidant enzyme activity, including peroxidase (POD), catalase (CAT), and polyphenol oxidase (PPO) was estimated in reference to [28, 29] and [30], respectively. Total phenols in the plant leaves were determined using the Folin-Ciocalteu assay [31]. Eight samples were applied for each treatment. Lipid peroxidation was estimated as malondialdehyde (MDA) in the plant leaf [32].

### Ultrastructural examination

Fourteen dpi, the plant leaves were sampled from each treatment for ultrastructural examinations [33]. The plant tissue was placed in aluminum planchets containing 15% dextran: water (w/v) and frozen in a high-pressure freezer. The frozen sample was placed in liquid nitrogen and kept in a plastic vial containing 2% OsO_4_ in acetone and 0.05% uranyl acetate. The vial was frozen at − 80 °C (3 days) in a freezer, transferred to a − 20 °C freezer (3 h), kept at 4 °C (2 h), and finally placed at room temperature. The plant tissue was washed using 100% acetone and then embedded in Spurr’s resin. Ultrathin sections were prepared using an ultramicrotome, collected on copper grids, stained with 2% uranyl acetate and then lead citrate, and finally examined using a transmission electron microscope (TEM) (JEOL 100-S TEM, Tokyo, Japan).

### Effect of infection on the photosynthetic pigments

Fourteen dpi and from each treatment, the second upper leaves were sampled and subjected to estimation of their photosynthetic pigments [34]. Three replicates of each treatment were analyzed.

### Assessment of the plant growth

Two weeks post inoculation, 10 plants were sampled from each treatment to evaluate the plant height, shoot and root dry weight, and leaf area.

### Field experiment

The field experiment was conducted at the experimental farm of El-Noubaria Agricultural Station, El-Behera, Egypt during two growing seasons (15 December to 30 April) 2020/2021 and 2021/2022. The experimental land was plowed, laser leveled, and divided into six plots. Each plot was 3 m^2^ containing 3 rows with 25 cm interspacing. Superphosphate and azote fertilizers were added to the experimental land at 0.06 and 0.32 units, respectively. Each row was sown by 5 g wheat grains. A well-known susceptible wheat cultivar (*T. spelta* L.) was planted at the border of the experiment to serve as a spreader. As a control treatment, plots containing 50 days-old plants of each cultivar were sprayed four times at 0.4 mL L^− 1^ and at two weeks intervals with the synthetic fungicide Crown^®^ (Propiconazole), El-Helb Pesticides & Chemicals Co, Egypt. To prepare the pathogen inoculum, 1 g of the fresh uredospores of *P. striiformis* was mixed with 20 g talcum powder. Artificial inoculation was carried out at the booting stage (92 days after planting) by dusting the pathogen inoculum on the spreader plants at evening to provide an enough humidity period for uredospores germination. The experiment was carried out in triplicates in a complete randomized block design. The applied treatments were as follows: G11: Gemmieza-11 sprayed with fungicide, G11 + P: infected Gemmieza-11, S1: Shandweel-1 sprayed with fungicide, S1 + P: infected Shandweel-1, M3: Misr-3 sprayed with fungicide, and M3 + P: infected Misr-3.

Fifteen dpi, severity of yellow rust was evaluated as previously mentioned. Area under the disease progress curve (AUDPC) was determined in reference to Pandey et al. [35] as a measure of the quantitative yellow rust resistance using the following equation:

$${\rm{AUDPC = D \times [}}\left( {{{{{\rm{Y}}_1}{\rm{ + }}{{\rm{Y}}_{\rm{k}}}} \over {\rm{2}}}} \right){\rm{ + }}{{\rm{Y}}_2}{\rm{ + }}{{\rm{Y}}_3}{\rm{ \ldots \ldots \ldots }}{{\rm{Y}}_{{\rm{k}} - 1}}{\rm{]}}$$

Where D = days between the records, Y_1_ = first disease record, Y_2_ = second disease record …. and Y_k_ = last disease record. At maturity, the yield parameters (grain yield and 1000 kernel weight) were estimated for each plot. The weights were measured after oven drying (75 °C) for 2 days.

At maturity, yield parameters including grain yield (kg) and 1000 kernel weight (g) in each treatment were evaluated and the loss (%) in each parameter was calculated.

### Statistical analyses

The obtained results were statistically analyzed *via* the statistical software CoStat version 6.45. Data were first tested for normality and then subjected to analysis of variance (ANOVA). Data means were compared using Tukey’s HSD test at *p* ≤ 0.05.

## Results

### Greenhouse evaluation of yellow rust severity

**Fig. 1:**
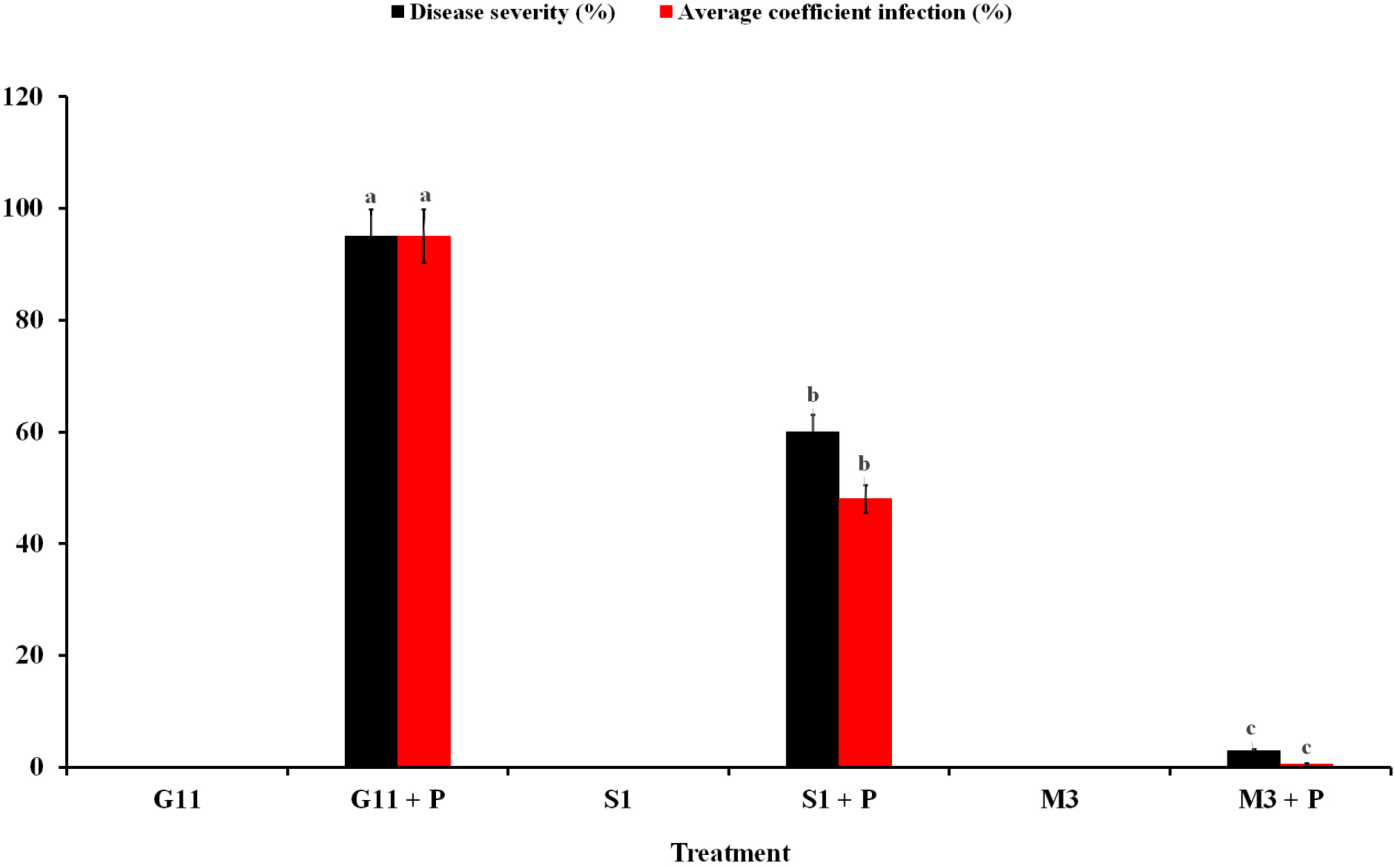
A bar chart demonstrates severity of yellow rust in three wheat *cvs*. Gemmieza 11, Shandweel-1, and Misr-3 in response to infection with *Puccinia striiformis* f.sp. *tritici* under the greenhouse conditions at 15 days after inoculation. Columns of each parameter superscripted by different letter are significantly different according to Tukey’s HSD test (*P* ≤ 0.05), each value is the mean of twelve samples. Error bars represent the standard deviations. Where, G11: non-infected Gemmieza-11, G11 + P: infected Gemmieza-11, S1: non-infected Shandweel-1, S1 + P: infected Shandweel-1, M3: non-infected Misr-3, and M3 + P: infected Misr-3

Yellow rust severity in the three tested wheat cultivars in response to inoculation with *P. striiformis* under greenhouse conditions is illustrated in Fig. 1. Results revealed that all wheat plants that were not inoculated did not express any symptoms of the disease. Conversely, all wheat cultivars inoculated with the pathogen inoculum showed varied disease severities. The highest yellow rust severity was observed in the wheat treatment (G11 + P) recording 95% severity and ACI. The wheat treatment (S1 + P) showed 60% severity, and 48% ACI, while the lowest severity of yellow rust disease (3%) was recorded in the (M3 + P) treatment. No significant ACI was recorded for the infected wheat plants *cv.* Misr-3 (Supplementary Figure).

### Transcriptional expression of the yellow rust-resistance genes

**Fig. 2:**
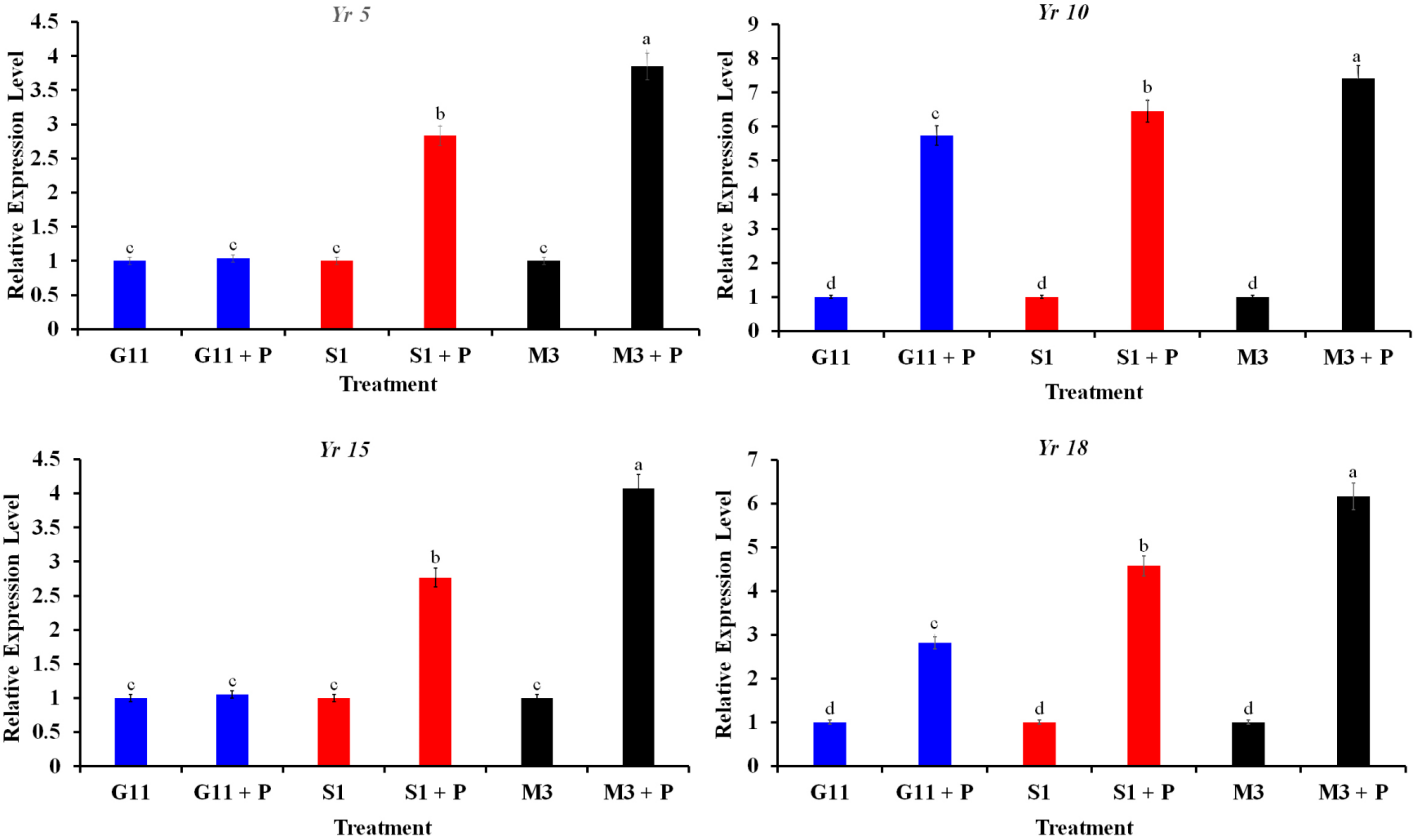
Bar charts demonstrate the transcriptional expression of the yellow rust-resistance genes (*Yr5*, *Yr10*, *Yr15*, and *Yr18*) in response to infection with *Puccinia striiformis* f.sp. *tritici* under the greenhouse conditions at 4 days after inoculation. Columns of each parameter superscripted by different letter are significantly different according to Tukey’s HSD test (*P* ≤ 0.05), each value is the mean of twelve samples. Error bars represent the standard deviations. Where, G11: Gemmieza-11 sprayed with fungicide, G11 + P: infected Gemmieza-11, S1: Shandweel-1 sprayed with fungicide, S1 + P: infected Shandweel-1, M3: Misr-3 sprayed with fungicide, and M3 + P: infected Misr-3

Transcriptional expression of the yellow rust-resistance genes (*Yr5*, *Yr10*, *Yr15*, and *Yr18*) upon infection with *P. striiformis* under the greenhouse conditions is presented in Fig. 2. Results from qPCR demonstrated that no significant difference was recorded for expression of *Yr5* between the wheat plants (*cv*. Gemmieza-11) inoculated with the *P. striiformis* and those non-inoculated. In contrast, inoculation of the wheat plants (*cv*. Shandweel-1) with *P. striiformis* overexpressed *Yr5* (2.84-fold), compared to the non-inoculated plants. However, the highest expression of *Yr5* gene (3.85-fold) was observed for the wheat plants *cv*. Misr-3 that were inoculated with *P. striiformis*, compared to the non-inoculated plants. Regarding *Yr10*, results showed that inoculation with *P. striiformis* significantly upregulated this gene in all tested wheat cultivars, compared to the non-inoculated plants. Upregulation of *Yr10* due to yellow rust infection was recorded as 5.74, 6.45, and 7.41-fold for the wheat plants *cv*. Gemmieza-11, Shandweel-1, and Misr-3, respectively. The observed results expressed the non-existence of a significant difference in expression of *Yr15* in wheat plants *cv*. Gemmieza-11 upon infection with yellow rust, compared to the non-inoculated plants. While, inoculation with *P. striiformis* considerably triggered expression of this gene, recording 2.76- and 4.07-fold in wheat plants of *cv*. Shandweel-1 and Misr-3, respectively. Infection with yellow rust overexpressed *Yr18* in all tested wheat cultivars, compared to the non-infected wheat plants, recording 2.81-, 4.58-, and 6.16-fold in wheat *cvs*. Gemmieza-11, Shandweel-1, and Misr-3, respectively.

### Biochemical defense responses in wheat plants

Activity of the tested antioxidant enzymes; mainly POD, PPO, and CAT, total phenol contents, and level of lipid peroxidation in wheat leaves of the three tested cultivars in response to inoculation with *P. striiformis* under greenhouse conditions is presented in Table 2. The obtained results indicated that inoculation with *P. striiformis* significantly induced activity of POD in wheat plants of the three tested cultivars. However, the greatest activity of POD was recorded in the treatment (M3 + P), recording 66.32 unit min^− 1^g^− 1^ f. wt, compared to the non-inoculated plants (45.3 unit min^− 1^g^− 1^ f. wt), followed by the infected wheat plants (S1 + P), which recorded (53.27 unit min^− 1^g^− 1^ f. wt), compared to the non-inoculated plants (31.83 unit min^− 1^g^− 1^ f. wt). The lowest activation was noticed for the wheat treatment (G11 + P). Regarding PPO, results demonstrated that inoculation with *P. striiformis* significantly triggered this enzyme activity in the three tested wheat cultivars at varied degrees. The greatest PPO activity (17.02 unit min^− 1^g^− 1^ f. wt) was recorded in the wheat plants *cv*. Misr-3, compared to the non-inoculated plants (7.96 unit min^− 1^g^− 1^ f. wt), followed by wheat plants *cv*. Shandweel-1. The lowest increment in PPO activity was observed in the wheat plants *cv*. Gemmieza-11, compared to the control plants (5.60 unit min^− 1^g^− 1^ f. wt). Results indicated that infection with yellow rust significantly induced activity of CAT in wheat plants *cvs*. Shandweel-1 and Misr-3, recording 35.37 and 23.40 unit min^− 1^g^− 1^ f. wt. While, no significant difference was observed in wheat *cv*. Gemmieza-11, compared to the control plants.

**Table 2:** Activity of peroxidase (POD), polyphenol oxidase (PPO), and catalase (CAT), total content of phenols, and lipid peroxidation level in the tested wheat leaves (*cvs*. Gemmieza 11, Shandweel-1, and Misr-3) in response to infection with *Puccinia Striiformis* f. sp. *tritici* at 15 days after inoculation*

Treatment	POD (Unit min^− 1^g^− 1^ f. wt)	PPO (Unit min^− 1^g^− 1^ f. wt)	CAT (Unit min^− 1^g^− 1^ f. wt)	Phenolic content (mg.100 g^− 1^ f. wt)	Lipid peroxidation (µmol MDA g^− 1^ f. wt)
G11	25.27 ± 2.09^d^	5.60 ± 0.35^d^	12.03 ± 2.23^d^	758.51 ± 10.86^e^	3.41 ± 0.36^c^
G11 + P	46.33 ± 4.18^c^	7.93 ± 1.39^c^	15.97 ± 2.74^cd^	861.44 ± 7.28^de^	12.97 ± 0.55^a^
S1	31.83 ± 7.51^dc^	7.13 ± 1.32^c^	15.17 ± 1.04^cd^	933.33 ± 18.37^d^	3.40 ± 0.30^c^
S1 + P	53.27 ± 3.62^b^	11.57 ± 1.22^b^	23.40 ± 1.95^b^	1290.01 ± 26.06^b^	10.03 ± 0.85^b^
M3	45.30 ± 3.94^c^	7.96 ± 1.14^c^	22.77 ± 4.45^b^	1073.36 ± 30.85^c^	3.43 ± 0.16^c^
M3 + P	66.32 ± 2.42^a^	17.02 ± 1.19^a^	35.37 ± 3.46^a^	1546.67 ± 45.33^a^	4.03 ± 0.15^c^

*Values, in each column, followed by different letter are significantly different according to Tukey’s HSD test (*P* ≤ 0.05), each value is the mean of eight samples ± SD. Where, G11: non-infected Gemmieza-11, G11 + P: infected Gemmieza 11, S1: non-infected Shandweel 1, S1 + P: infected Shandweel 1, M3: non-infected Misr-3, and M3 + P: infected Misr-3

The obtained results from this study indicated the inducing effect of yellow rust infection on phenolic contents of the wheat *cvs*. Shandweel-1 and Misr-3, recording 1546.67 and 1290.01 mg 100 g^− 1^ f. wt; respectively, compared to their non-infected controls. In contrast, no significant difference was observed between the infected and non-infected wheat plants *cv*. Gemmieza-11 in this regard. The recorded results displayed that infection with yellow rust caused a considerable increment in the lipid peroxidation level in the wheat plants *cvs*. Gemmieza-11 and Shandweel-1, recording 12.97 and 10.03 µmol MDA g^− 1^ f. wt, respectively, compared to their non-infected controls. On the contrary, there was no significant difference observed between the infected and non-infected wheat plants *cv*. Misr-3 in this regard.

### Effect of infection with yellow rust on the leaf ultrastructure

**Fig. 3:**
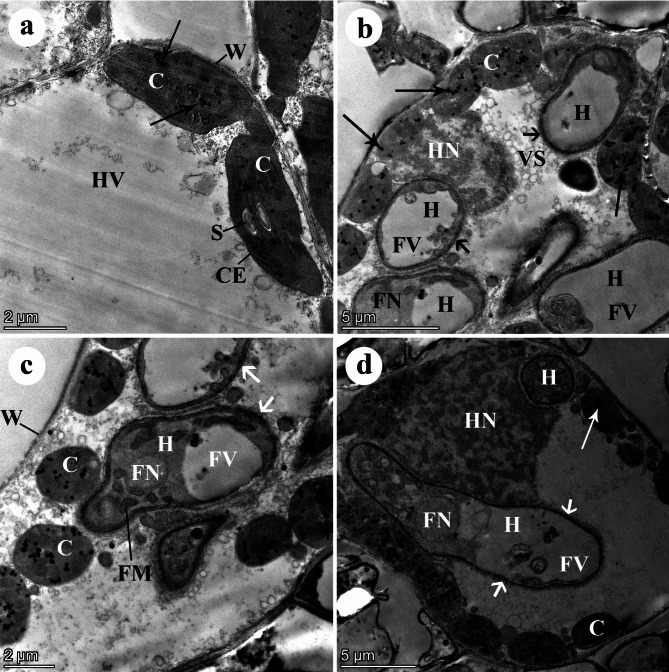
TEM micrographs of mesophyll cells of a highly susceptible wheat *cv*. Gemmieza-11. (**A**) Healthy (non-infected) host mesophyll cells showing normal ellipsoidal chloroplasts (C). The chloroplasts represent an organized membrane system of grana and intergranal lamellae. Note starch grains (S), a few electron-dense plastoglobuli (arrows), an intact chloroplast envelope (a big cell vacuole) (HV), and a thin host cell wall (W). Bar = 2 μm. (**B**) Infected cell showing many haustoria (H). Note abnormal globoid chloroplasts (C) with a non-organized membrane system of grana and intergranal lamellae, numerous electron-dense plastoglobuli (arrows), and an abnormally affected host nucleus (N). The haustoria are enclosed by electron-dense haustorial walls (arrowheads) and contain big vacuoles (FV). Note also the numerous vesicles (VS) inside the cell vacuole. Bar = 5 μm. (**C**) Infected cell showing many haustoria (H). Note abnormal spherical chloroplasts (C) with a non-organized membrane system of grana and intergranal lamellae, numerous electron-dense plastoglobuli (arrows). The chloroplasts are moved away from the cell wall (W). The haustoria are enclosed by electron-dense haustorial walls (arrowheads) and contain vacuoles (FV), nucleus (FN), and mitochondria (FM). Bar = 2 μm. (**D**) Infected cell showing two haustoria (H). Note abnormally degenerated chloroplasts (C) with a non-organized membrane system of grana and intergranal lamellae, numerous electron-dense plastoglobuli (arrows). The haustoria are enclosed by electron-dense haustorial walls (arrowheads) and contain vacuoles (FV), and nucleus (FN). Note also that an abnormal host nucleus (HN) is nearly curved around one haustorium. Bar = 5 μm

Using TEM, examining the chloroplasts of the non-infected wheat mesophyll cells demonstrated normal ellipsoidal chloroplasts. The chloroplasts represent an organized membrane system of grana and inter-grana lamellae. A few grains of starch, electron-dense plastoglobuli, and intact chloroplast envelopes were also observed. On the contrary, the infected mesophyll cells of the highly susceptible wheat *cv*. Gemmieza-11 showed many haustoria and abnormal globoid chloroplasts with a non-organized membrane system of grana and inter-grana lamellae. Numerous electron-dense plastoglobuli were present inside the chloroplasts, and an abnormally affected host nucleus was also detected. The haustoria were enclosed by electron-dense haustorial walls and contained big vacuoles, nuclei, and mitochondria. Rarely, the chloroplasts moved away from the cell wall. In some cases, abnormally degenerated chloroplasts were observed. Surprisingly, a nucleus was curved around a single haustorium (Fig. 3).

**Fig. 4:**
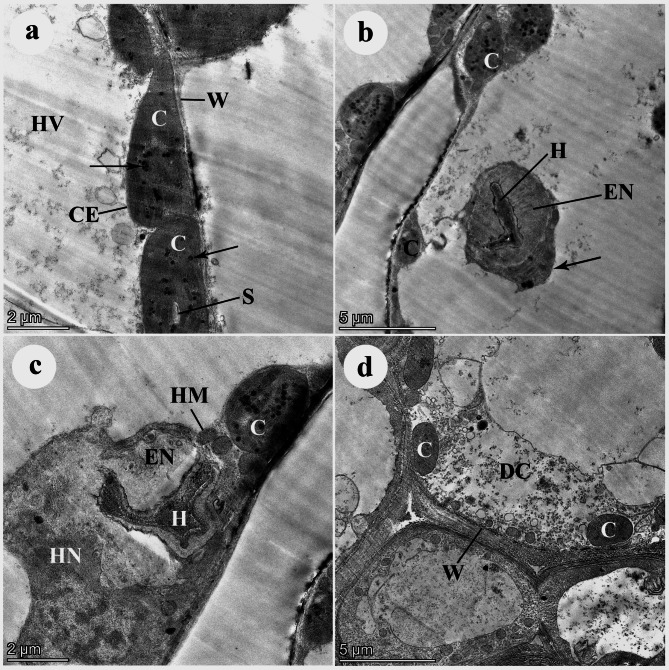
TEM micrographs of mesophyll cells of a moderately susceptible wheat *cv*. Shandawel. (**A**) Healthy (non-infected) host mesophyll cells showing normal ellipsoidal chloroplasts (C). The chloroplasts represent an organized membrane system of grana and intergranal lamellae. Note a starch grain (S), few electron-dense plastoglobuli (arrows), an intact chloroplast envelope (CE), a big cell vacuole (HV), and a thin host cell wall (W). Note also that the chloroplasts are closely associated with the host cell wall. Bar = 2 μm. (**B**) Infected host cell show a highly degenerated haustorium (H). The necrotic haustorium is surrounded by a wide electron-lucent matrix, or encasement (EN). Note collapsed abnormal chloroplasts (C) with non-organized systems of grana and intergranal lamellae. Note also the associated chloroplast (arrow) with the necrotic haustorium. Bar = 0.5 μm. (**C**) Infected host cell show a highly degenerated haustorium (H). The necrotic haustorium is surrounded by a wide an electron-lucent matrix, or encasement (EN). Note abnormal spherical chloroplast (C) with non-organized systems of grana and intergranal lamellae. Note also an abnormal host nucleus (HN) and host mitochondria (HM). Bar = 2 μm. (**D**) The infected host cell shows a degenerated granulated cytoplasm (DC) containing a few small chloroplasts (C) with non-organized systems of grana and intergranal lamellae. Not a thick host cell wall (W). Bar = 0.5 μm

In the case of the moderately susceptible wheat *cv*. Shandweel-1, healthy (non-infected) host mesophyll cells showed normal ellipsoidal chloroplasts. The chloroplasts represent an organized membrane system of grana and inter-grana lamellae starch grains, a few electron-dense plastoglobuli, an intact chloroplast envelope, a big cell vacuole, and a thin host cell wall. The chloroplasts were firmly associated with cell wall of the host. Conversely, the infected host cells displayed a highly degenerated haustorium, and a wide encasement or electron-lucent matrix was surrounding the necrotized haustorium. The host cell nucleus seemed to be abnormal. Furthermore, the infected host cell showed a degenerated granulated cytoplasm and a thick host cell wall (Fig. 4).

**Fig. 5:**
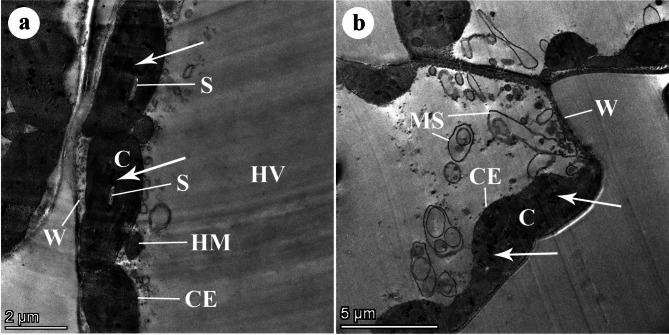
TEM micrographs of mesophyll cells of a highly resistant wheat *cv*. Misr 3. (**A**) Healthy (non-infected) host mesophyll cells showing normal ellipsoidal chloroplasts (C). The chloroplasts represent an organized membrane system of grana and intergranal lamellae. Note starch grains (S), a few electron-dense plastoglobuli (arrows), an intact chloroplast envelope (CE), a big cell vacuole (HV), and a thin host cell wall (W). Note also that the chloroplasts are closely associated with the host cell wall and host mitochondria (HM). Bar = 2 μm. (**B**) Host cells from inoculated highly resistant wheat cultivars. The haustoria are not found inside the host cell. Host mesophyll cells show normal ellipsoidal chloroplasts (C). The chloroplasts represent an organized membrane system of grana and intergranal lamellae. Note a few electron-dense plastoglobuli (arrows), an intact chloroplast envelope (CE), and a big cell vacuole (HV) containing membranous structures (MS). Note also that the chloroplasts are closely associated with the host cell wall (W). Bar = 5 μm

In regard to the highly resistant wheat r *cv*. Misr-3, no haustoria were observed inside the mesophyll cells. Healthy (non-infected) host mesophyll cells showed normal ellipsoidal chloroplasts. The chloroplasts represent an organized membrane system of grana and inter-grana lamellae with grains of starch and a few electron-dense plastoglobuli. The chloroplast envelope was intact. The mesophyll cell was characterized by a big cell vacuole and a thin wall. The chloroplasts were firmly associated with the cell wall of the host and host mitochondria (Fig. 5).

### Effect of infection with yellow rust on the photosynthetic pigments

Contents of the photosynthetic pigments in wheat leaves of the tested cultivars in response to infection with yellow rust are shown in Table 3. The obtained results demonstrated that infection with yellow rust caused significant reduction of the photosynthetic pigments, mainly Chl. *a* and *b* and carotenoids in wheat leaves of *cvs*. Gemmieza-11 and Shandweel-1 compared to their non-infected control plants. No significant differences were observed between the infected *cv*. Misr-3 and the non-infected wheat plants in this regard. In the infected wheat cultivars, the highest reduction (61.75%) in total photosynthetic pigments was recorded in *cv*. Gemmieza-11, and followed by that of *cv*. Shandweel-1 (29.43%). Meanwhile, the least reduction was observed for *cv*. Misr-3 (8.88%).

**Table 3:** Contents of the photosynthetic pigments in the tested wheat leaves (*cv*. Gemmieza 11, Shandweel-1, and Misr-3) in response to infection with *Puccinia Striiformis* f. sp. *tritici* at 14 days after inoculation*

Treatment	Chl. a (mg g^− 1^ f. wt)	Chl. b (mg g^− 1^ f. wt)	Carotenoids (mg g^− 1^ f. wt)	Total pigments (mg g^− 1^ f. wt)
G11	1.354 ± 0.109^c^	0.795 ± 0.019^c^	0.378 ± 0.025^b^	2.527 ± 0.357^c^
G11 + P	0.802 ± 0.179^d^	0.483 ± 0.025^d^	0.278 ± 0.131^c^	1.563 ± 0.177^d^
S1	2.652 ± 0.691^a^	2.132 ± 0.048^a^	0.397 ± 0.057^b^	5.181 ± 0.641^a^
S1 + P	1.669 ± 0.185^b^	1.625 ± 0.025^b^	0.362 ± 0.051^b^	3.656 ± 0.954^b^
M3	2.694 ± 0.176^a^	0.719 ± 0.039^c^	0.437 ± 0.074^a^	3.850 ± 0.640^b^
M3 + P	2.396 ± 0.109^ab^	0.676 ± 0.014^c^	0.436 ± 0.061^a^	3.508 ± 0.331^b^

*Values, in each column, followed by different letter are significantly different according to Tukey’s HSD test (*P* ≤ 0.05), each value is the mean of three samples ± SD. Where, G11: non-infected Gemmieza-11, G11 + P: infected Gemmieza 11, S1: non-infected Shandweel 1, S1 + P: infected Shandweel 1, M3: non-infected Misr-3, and M3 + P: infected Misr-3

### Effect of infection with yellow rust on the wheat growth

Growth parameters of the tested wheat cultivars in response to infection with yellow rust are shown in Table 4. The results expressed that infection with yellow rust significantly reduced the wheat plant height in *cv*. Gemmieza-11 by 16.6%, compared to its non-infected control plants, however, there was no significant difference observed in the wheat plant height between the infected and non-infected plants *cvs*. Shandweel-1 and Misr-3. Regarding the shoot and root dry weights; the results indicated that infection with yellow rust reduced both tested parameters of wheat plants of *cv*. Gemmieza-11 and Shandweel-1, compared to their non-infected controls. While, no significant effect was recorded in this regard between the infected and non-infected plants of *cv*. Misr-3. However, the reducing effect was higher in wheat plants of *cv*. Gemmieza-11 than in those of *cv*. Shandweel-1. At the same time, results from this study demonstrated that infection with yellow rust reduced the leaf area of the wheat plants of the three tested cultivars; however, the reduction in *cv*. Gemmieza-11 was the highest, followed by *cv*. Shandweel-1, while the lowest reduction was observed in *cv*. Misr-3.

**Table 4:** Growth parameters of the tested wheat cultivars (*cv*. Gemmieza 11, Shandweel-1, and Misr-3) in response to infection with *Puccinia Striiformis* f. sp. *tritici* at 14 days after inoculation*

Treatment	Plant height (cm)	Shoot dry weight (g)	Root dry weight (g)	Leaf area (cm^2^)
G11	56.33 ± 3.06^ab^	2.23 ± 0.37^ab^	0.39 ± 0.053^a^	18.10 ± 0.70^b^
G11 + P	47.00 ± 1.12^c^	1.23 ± 0.21^c^	0.17 ± 0.045^c^	11.30 ± 0.97^d^
S1	64.33 ± 1.53^a^	2.52 ± 0.38^a^	0.33 ± 0.095^b^	21.65 ± 1.55^a^
S1 + P	60.00 ± 2.20^ab^	1.63 ± 0.21^bc^	0.21 ± 0.030^c^	15.50 ± 0.98^c^
M3	56.33 ± 3.21^ab^	2.27 ± 0.35^ab^	0.40 ± 0.041^a^	19.70 ± 0.81^ab^
M3 + P	55.33 ± 5.85^bc^	2.15 ± 0.34^ab^	0.37 ± 0.057^ab^	15.53 ± 1.12^c^

*Values, in each column, followed by different letter are significantly different according to Tukey’s HSD test (*P* ≤ 0.05), each value is the mean of ten samples ± SD. Where, G11: non-infected Gemmieza-11, G11 + P: infected Gemmieza 11, S1: non-infected Shandweel 1, S1 + P: infected Shandweel 1, M3: non-infected Misr-3, and M3 + P: infected Misr-3

### Disease evaluation under field conditions

Field evaluation of yellow rust severity and AUDPC in the three tested wheat cultivars in two growing seasons is presented in Table 5. The results indicated that all protected plants of the three tested wheat cultivars that were sprayed with the fungicide showed no infection symptoms. On the contrary, the three wheat cultivars that were inoculated with the pathogen showed, at varying degrees, typical symptoms of yellow rust. The highest severity was observed in the wheat plants of *cv*. Gemmieza-11, recording 73.33 and 53.33% in the growing seasons 2020/2021 and 2021/2022, respectively. While the lowest severity was observed in the wheat plants of *cv*. Misr-3, recording 6.67 and 3.00% in the growing seasons 2020/2021 and 2021/2022, respectively. Regarding AUDPC, the wheat plants of *cv*. Gemmieza-11 recorded the highest value of 1333.33 and 1016.67 in the growing seasons 2020/2021 and 2021/2022, respectively. The lowest value was recorded for the wheat plants *cv*. Misr-3 (25.00 and 15.00) in the growing seasons 2020/2021 and 2021/2022, respectively.

**Table 5:** Field evaluation of yellow rust severity and area under the disease progress curve (AUDPC) in three wheat cultivars (*cv*. Gemmieza 11, Shandweel-1, and Misr-3) in two growing seasons (2020/2021 and 2021/2022)*

Treatment	2020/2021		2021/2022	
	Disease severity (%)	AUDPC	Disease severity (%)	AUDPC
G11	0.0^c^	0.0^c^	0.0^c^	0.0^c^
G11 + P	73.33 ± 5.77^a^	1333.33 ± 152.75^a^	53.33 ± 5.77^a^	1016.67 ± 125.83^a^
S1	0.0^c^	0.0^c^	0.0^c^	0.0^c^
S1 + P	53.33 ± 5.77^b^	1016.67 ± 125.83^b^	43.33 ± 5.77^b^	608.33 ± 76.38^b^
M3	0.0^c^	0.0^c^	0.0^c^	0.0^c^
M3 + P	6.67 ± 2.89^c^	25.00 ± 5.00^c^	3.00 ± 1.73^c^	15.00 ± 4.66^c^

*Values, in each column, followed by different letter are significantly different according to Tukey’s HSD test (*P* ≤ 0.05), each value is the mean of three plots ± SD. Where, G11: Gemmieza-11 sprayed with fungicide, G11 + P: infected Gemmieza 11, S1: Shandweel 1 sprayed with fungicide, S1 + P: infected Shandweel 1, M3: Misr-3 sprayed with fungicide, and M3 + P: infected Misr-3

### Effect of infection with yellow rust on wheat yield

Field evaluation of yield parameters in the three tested wheat cultivars in the two growing seasons (2020/2021 and 2021/2022) in response to infection with yellow rust is shown in Table 6. The results revealed that infection with yellow rust considerably reduced the grain yield in both *cvs*. Gemmieza-11 and Shandweel-1, compared to the protected wheat plants (sprayed with the fungicide) recording 50.76%, 24.76% losses in the growing season 2020/2021; respectively, and 44.60% 18.48% losses in in the growing season 2021/2022, respectively. In contrast, in both growing seasons, there was no significant difference recorded between grain yield of the infected and protected wheat plants of *cv*. Misr-3. In both growing seasons, the highest grain yield was observed in wheat plants of *cv*. Misr-3, whether infected or non-infected with yellow rust. At the same time, infection with yellow rust reduced the 1000 kernel weight in all tested cultivars, compared to the protected wheat plants *cvs*. Gemmieza-11, Shandweel-1, and Misr-3, recording 50.99%, 19.37%, and 2.22% losses, respectively, in the growing season 2020/2021. Meanwhile, in the growing season 2021/2022, the wheat *cvs*. Gemmieza-11 and Shandweel-1 recorded 41.67% and 15.51% losses in the 1000 kernel weight, while no significant effect was observed for infection with yellow rust on the wheat *cv*. Misr-3 in this regard.

**Table 6:** Field evaluation of yield parameters in three wheat cultivars (*cvs*. Gemmieza 11, Shandweel-1, and Misr-3) at two growing seasons (2020/2021 and 2021/2022) in response to infection with yellow rust*

Treatment	2020/2021				2021/2022			
	Grain yield (kg)	Losses (%)	1000 kernel weight (g)	Losses (%)	Grain yield (kg)	Losses (%)	1000 kernel weight (g)	Losses (%)
G11	27.82 ± 1.44^b^	0^d^	56.86 ± 0.18^a^	0^d^	28.73 ± 0.81^b^	0^d^	56.94 ± 0.14^a^	0^d^
G11 + P	13.70 ± 1.80^d^	50.76 ± 0.55^a^	27.87 ± 0.15^d^	50.99 ± 0.37^a^	15.92 ± 0.53^e^	44.60 ± 1.65^a^	33.21 ± 0.34^d^	41.67 ± 0.55^a^
S1	26.92 ± 1.62^b^	0^d^	50.37 ± 0.77^b^	0^d^	27.54 ± 1.40^c^	0^d^	51.60 ± 027^b^	0^d^
S1 + P	20.25 ± 1.74^c^	24.76 ± 1.93^b^	40.61 ± 0.52^c^	19.37 ± 0.29^b^	22.45 ± 1.38^d^	18.48 ± 0.97^b^	43.60 ± 0.23^c^	15.51 ± 0.37^b^
M3	31.02 ± 2.02^a^	0^d^	57.01 ± 1.77^a^	0^d^	31.13 ± 1.12^a^	0^d^	55.27 ± 2.17^a^	0^d^
M3 + P	30.21 ± 1.17^a^	3.85 ± 0.69^c^	50.37 ± 0.76^b^	2.22 ± 0.43^c^	30.18 ± 1.04^a^	3.05 ± 0.59^c^	54.77 ± 1.74^ab^	1.21 ± 0.29^c^

*Values, in each column, followed by different letter are significantly different according to Tukey’s HSD test (*P* ≤ 0.05), each value is the mean of three plots ± SD. Where, G11: Gemmieza-11 sprayed with fungicide, G11 + P: infected Gemmieza 11, S1: Shandweel 1 sprayed with fungicide, S1 + P: infected Shandweel 1, M3: Misr-3 sprayed with fungicide, and M3 + P: infected Misr-3

## Discussion

Extensive research studies have been conducted to develop new wheat genotypes that are resistant to *P. striiformis* due to breakdown of resistance in many wheat cultivars. In Egypt, several cultivars such as Chenab-70, Giza-139, Mexipak-69, and Super-X have been eliminated due to their sensitivity [20]. In contrast, the emergence of new virulent races of this fungus worldwide revealed its rapid evolution under different climatic and environmental conditions [18]. Particularly, since *Berberis* and *Mahonia* spp. have been recognized as alternative hosts of this fungus, researchers have recently concentrated their studies on the possibility of emergence of new virulent races *via* sexual reproduction [36]. In the present study, all wheat cultivars inoculated with uredospores of *P. striiformis* showed varied levels of yellow rust severity, indicating different degrees of disease resistance. This resistance variability was correlated with varied upregulation levels of several rust-resistance genes *Yr5*, *Yr10*, *Yr15*, and *Yr18* and varied triggering of multiple defense responses. In agreement with the previous results reported by Esmail et al. [37] on *Lr50* gene of wheat cultivars inoculated with *P. triticina*, the present study showed a rust-resistance correlation with the overexpression of the *Yr5*, *Yr10*, *Yr15*, and *Yr18* genes in the resistant *cv*. Misr-3, followed by the moderately sensitive *cv*. Shandweel-1, while no difference in the expression of these genes was recorded in the infected plants of *cv*. Gemmieza-11, when compared to the non-infected ones. The obtained results demonstrated that both *Yr5* and *Yr15* were more effective genes than *Yr10* and *Yr18* with regard to rust resistance. The obtained results showed that infection with stripe rust upregulated *Yr10* and *Yr18* in the three tested cultivars although there is quite a difference in cultivars’ resistance degrees. In contrast, the expression of *Yr5* and *Yr15* in the three cultivars was correlated with the varied resistance levels, where they were overexpressed in the resistant cultivars and not expressed in the susceptible one. This result is in agreement with that obtained by Ragab et al. [38] who tested the efficiency of *Yr5*, *Yr10*, *Yr15* and *YrSp* in the stripe rust resistance on two wheat cultivars Sids 12 and Gemmeiza 11. He arranged the four tested genes with regard to the stripe rust resistance as follows *Yr5 >Yr15 >YrSp >Yr10.* The association between expression of *Yr5* and *Yr15* and cultivars resistance degrees may indicate their effective role(s) in this concern. In a recent study investigating the presence of *Yr5*, *Yr10*, *Yr15*, and *Yr24/Yr26* genes in 38 highly-resistant wheat genotypes, 14 lines were found to have one gene, 16 lines carry two genes, and 7 lines had three gene combinations [17]. This result indicates the crucial role of these genes in yellow rust resistance and are still effective against this pathogen. In contrast, other *Yr* genes such as *Yr2-4*, *Yr6-9*, *Yr17-19*, *Yr21-27*, and *YrA* have become ineffective in this regard due to the continuous and fast evolution of *P. striiformis* [17]. Esmail et al. [39] reported *P. striiformis* f. sp. *tritici* (race 151E80), which was used in this study, as one of 6 aggressive races that were virulent on *Yr1*, *Yr2*, *Yr6*, *Yr7*, *Yr8*, *Yr9*, *Yr10*, *Yr19*, *Yr22*, *Yr23*, *Yr25*, *YrCle*, *YrMor*, and *YrSP* recording an aggressiveness of 58.33%. Presence of more than one *Yr* gene is important to develop cultivars with durable resistance [40]. However, wheat resistance is not limited to these genes, but each *Yr* gene regulates many other defense-related genes that contribute to the final plant resistance [41]. In a recent microarray study conducted to investigate the genes contributing to *Yr5*-related resistance in wheat, 61 transcripts have been identified. These genes are involved in different signaling pathways and responsible for various defensive mechanisms and hypersensitive responses, including accumulation of PR- proteins, flavonoid, and polyphenolic compounds [42].

Accumulation of ROS is critical for the rust-fungus virulence, germination of its uredospores, and suppression of plant immunity [19]. However, wheat plants can eliminate or alleviate oxidative damage through a complex antioxidant system, which generates different enzymatic and non-enzymatic antioxidants [43]. In this regard, the obtained results in this study expressed higher activity of the three antioxidant enzymes POD, PPO, and CAT in the resistant *cv*. Misr-3, followed by the moderately sensitive *cv*. Shandweel-1 than in the sensitive *cv*. Gemmieza-11 upon infection with *P. striiformis*. These enzymes accumulate in the plant to protect the cells from ROS-induced oxidative bursts and/or scavenge the harmful free radicals [44, 45]. In addition, an increment in the level of lipid peroxidation was also recorded upon infection of wheat with *P. striiformis*, especially in the susceptible *cv*. Gemmieza-11 and moderately susceptible *cv*. Shandweel-1; however, the resistant *cv*. Misr-3 did not record a significant increase. Under biotic and abiotic stresses, ROS accumulation may cause lipid peroxidation, leading to oxidative damage of the plant cell membrane [46].

Phenolic compounds play valuable roles in plant development and defense against several biotic and abiotic stresses [46–48]. In the present study, yellow rust infection enhanced the phenolic contents of the wheat plants *cvs*. Shandweel-1 and Misr-3. Conversely, the susceptible *cv*. Gemmieza-11 had a lower content of these compounds, thus the pathogen infection may disrupt the metabolic potentials of the plant necessary for its development and growth. Accordingly, the susceptible cultivar becomes more vulnerable to pathogen multiplication. These results are in line with those of the recent study conducted by Rashad et al. [16], who revealed a noticeable downregulation of six of the studied polyphenol biosynthesis genes in the susceptible wheat *cv*. Sids12 during the early stage of infection by *P. striiformis*, indicating the importance of this action in obstructing the plant immunity.

In this study, TEM observations demonstrated that the infected mesophyll cells of the highly susceptible wheat *cv*. Gemmieza-11 had abnormal globoid chloroplasts with a non-organized membrane system, and electron-dense haustorial walls enclosing the haustoria. In case of the highly resistant wheat *cv*. Misr-3, no haustoria were observed inside the mesophyll cells and the chloroplast envelope was intact, indicating failure of infection and penetration processes due to the high defensive responses. Meanwhile, the moderately resistant wheat *cv*. Shandweel-1 displayed highly degenerated haustoria, a degenerated granulated cytoplasm, and a thick host cell wall. This result is in accordance with the previous findings of Esmail et al. [50]. Different hypersensitivity responses were observed in ultrastructure of the three studied wheat cultivars, which indicate the variability of the plant cell responses against the invading fungus. The number, size, and wall thickness of fungal haustoria in the infected cells of *cv*. Gemmieza-11 revealed high susceptibility to this pathogen and weakness of plant responses and immunity. The most noticeable damage was observed in the membrane system of the grana and the intergranal lamellae of the chloroplasts, leading to destruction of the photosynthetic system in the plant leaves, which explains the decline in contents of the photosynthetic pigments. These observed dis-organizations of the wheat susceptible *cv*. Gemmieza-11 cell organelles may be attributed to the existence of non-significant difference in expression of *Yr5* and *Yr15*, and upregulation of *Yr10* resistance genes, leading to reduced host cell resistance. This is in addition to lower antifungal phenolic contents recorded in this susceptible *cv*. Gemmieza-11 that allowed for haustorial penetration and subsequent growth and multiplication of the fungus. Moreover, the level of antioxidant enzymes (PR-proteins) such as chitinase may be low in this susceptible cultivar as currently recorded with respect to POD, PPO, and CAT levels, and thus allowed penetration of the fungal haustorium and flow of water and nutrients from the plant cell to the pathogen, which was followed by pathogen multiplication, leading to subsequent distortion of the host cell organelles observed through TEM micrographs. This result is in agreement with results of the previous study carried out by Chen et al. [51], who revealed extensive damages to the thylakoid membranes of the susceptible wheat cultivar, indicating that the thylakoid protein phosphorylation represents a host response to infection. In contrast, the highly degenerated haustorium with the surrounding electron-lucent matrix that was observed in infected cells of the moderately susceptible wheat cultivar indicated a moderate plant resistance level. This result may attributed to the fact that the haustorium succeeded in penetrating the cell, but its growth was restricted by several plant hypersensitivity reactions, including overexpression of *Yr5*, *Yr10*, *Yr15*, and *Yr18* genes that induced host cell resistance, cytoplasmic granulation, production of antioxidant enzymes such as chitinase that hydrolyzed the fungal haustorium, caused its degeneration as observed in the TEM micrographs, prevented its growth, and thus prevented fungal multiplication. These were in addition to the formation of antifungal phenolic compounds that inhibited the fungal growth, and accumulation of lignin that caused host cell wall thickening and surrounded the haustorium in the form of electron-lucent matrix; accordingly reduced growth of the fungal haustorium and prevented transfer of water and nutrients to the fungus.

In Egypt, the cool and moist conditions prevalent from December to April allow yellow rust to develop faster and allow abundant production of uredospores. Under field conditions, *P. striiformis* infection significantly increased the disease severity and AUDPC in the susceptible *cv*. Gemmieza-11, and followed by the moderately susceptible *cv*. Shandweel-1. However, a noticeable reduction in these parameters was observed in the resistant *cv*. Misr-3. Compared to several epidemiological parameters, several studies confirmed AUDPC as a convenient and more reliable estimator for measuring partial resistance (PR), which is attributed to the fact that during an epidemic, AUDPC represents the amount of rust infection and the rate of increase of this disease [52]. Accordingly, and for practical purposes, selection of cultivars with lower AUDPC is preferred. In this study, *cvs*. Shandweel-1 and Misr-3 displayed low AUDPC, thus are regarded as having high resistance to yellow rust.

In conclusion, this study showed variability of the wheat defensive responses to yellow rust based on the plant resistance level. The difference in the expression of *Yr5*, *Yr10*, *Yr15*, and *Yr18* indicated that *Yr5* and *Yr15* were more effective than *Yr10* and *Yr18* with regard to rust resistance. The association between expression of *Yr5* and *Yr15* and cultivars resistance degrees may indicate their effective role(s) in this concern. Results of several previous studies are in accordance with our findings concerning the effects of expression of the tested genes on inducing wheat cultivars resistance to stripe rust. Many greenhouse and field trials were conducted in a previous study [38] to enhance stripe rust resistance of two Egyptian wheat cultivars; mainly Sids 12 and Gemmieza-11 using four monogenic lines *Yr5*, *Yr10*, *Yr15*, and *Sp*. Results indicated that wheat genotypes carrying *Yr5* and *Yr15* genes at the seedling and adult stages expressed high resistance to *P. striiformis*. In Kazakhstan, among a collection of 70 wheat cultivars and breeding lines, three stripe rust resistance genes; mainly *Yr10*, *Yr5*, and *Yr15* displayed high frequencies of occurrence [53]. Moreover, at two locations in India and under field conditions, 13 known *Yr* gene-associated markers belonging to *Yr5*, *Yr10*, *Yr15*, and *Yr24/Yr26* were used to identify the genes in selected wheat cultivars that were resistant to stripe rust. Results demonstrated that *Yr5* existed in 16 lines, *Yr10* in 10 lines, and *Yr15* in 14 lines [12]. These genes play important roles in regulating different signaling pathways and are responsible for various defensive mechanisms and hypersensitive responses. However, contribution of more than one *Yr* gene is important to develop more resistant cultivars. Various defense responses were detected such as the significant increment of the fungi-toxic phenolic contents and enhanced activity of several antioxidant enzymes, including POD, PPO, and CAT, which acted as scavengers for ROS and free radicals.

## Electronic supplementary material

Below is the link to the electronic supplementary material.

Supplementary Material 1

## Data Availability

No datasets were generated or analysed during the current study.
