# Supplementary figures and images for: Host resistance responses against Puccinia striiformis f. sp. tritici in wheat cultivars with different resistance levels: molecular, biochemical, and ultrastructural studies
Source: BMC Plant Biol. 2024 Nov 28;24:1134. doi: 10.1186/s12870-024-05811-0 (PMC11603659; doi:10.1186/s12870-024-05811-0)

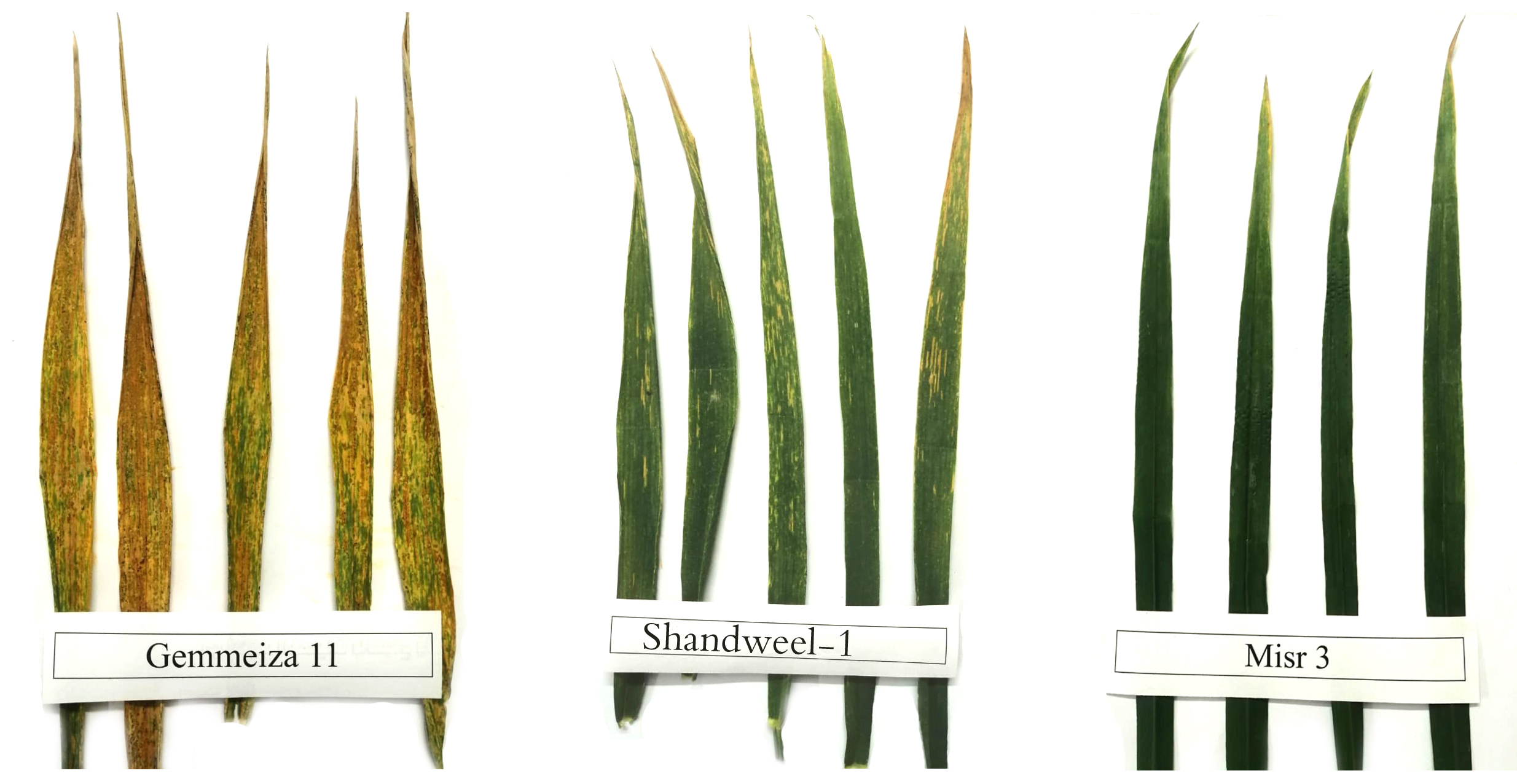

Supplement: Supplementary file 1 — Supplementary Material 1 [file 12870_2024_5811_MOESM1_ESM.jpg]
